# Development and Characterization of κ‐Carrageenan‐Based Edible Films Enriched With Resveratrol or Quercetin for Shelf‐Life Extension of Goat Meat Burger Patties

**DOI:** 10.1111/1750-3841.70277

**Published:** 2025-05-19

**Authors:** Ezgi Şengül, Damla Bilecen Şen

**Affiliations:** ^1^ Division of Food Engineering, Graduate School of Natural and Applied Sciences Burdur Mehmet Akif Ersoy University Burdur Türkiye; ^2^ Department of Food Engineering, Faculty of Engineering and Architecture Burdur Mehmet Akif Ersoy University Burdur Türkiye

**Keywords:** burger patty, edible film, goat meat, quercetin, resveratrol, shelf life, κ‐carrageenan

## Abstract

This study aimed to develop κ‐carrageenan‐based edible films enriched with resveratrol or quercetin and evaluate their potential in extending the shelf life and preserving the quality of goat meat burger patties during 14 days of storage at 4°C. The quercetin film solution exhibited greater antioxidant capacity than the resveratrol‐containing film, as indicated by higher total phenolic content (389.00 ± 0.42 mg GAE/g), DPPH inhibition (91.85 ± 0.06%), and FRAP values (545.08 ± 0.37 µmol Fe^2^⁺/L) (*p* < 0.05). Incorporation of either compound decreased L^*^ values and increased b^*^ values (*p* < 0.05); resveratrol reduced opacity, whereas quercetin enhanced it (*p* < 0.05). No inhibition zones were observed against *Listeria monocytogenes*, *Escherichia coli*, *Staphylococcus aureus*, or *Salmonella* Typhimurium (*p* > 0.05). SEM micrographs showed that both compounds improved the homogeneity of the film matrix. The chemical composition and water activity of the patties were not significantly affected by the treatment, storage day, or their interaction (*p* > 0.05). However, patties coated with the quercetin films had a lower pH (pH 6.18 ± 0.01) than the control (pH 6.27 ± 0.03) (*p* < 0.05) and retained their initial color better. Additionally, quercetin effectively delayed lipid oxidation (21.98 ± 1.94 µmol MDA/kg) and inhibited microbial growth, maintaining TMAB counts below 10 CFU/g by Day 14 (*p* < 0.05). Overall, quercetin‐containing κ‐carrageenan films demonstrated promising technological and preservative properties, offering a sustainable alternative to conventional packaging for heat‐treated ready‐to‐eat meat products. Future research should explore strategies such as controlled release, encapsulation, and synergistic bioactive combinations to further enhance efficacy.

## 1 Introduction

Meat and meat products are primary sources of animal protein, offering essential vitamins and minerals (Heinz and Hautzinger 2007). However, their high saturated fat and cholesterol contents have been linked to negative health effects (Klurfeld 2015; Wang et al. 2016). With increasing health awareness, there is a growing demand for healthier food options, making sustainable production of animal products essential (Thornton 2010; Henchion et al. 2014; Weber and Windisch 2017). Goat meat, with its lower fat content and favorable fatty acid profile, is emerging as a healthier alternative in the global meat industry (Tüfekci 2022).

Meat's high water activity and basic pH provide a favorable environment for microbial growth, while its lipid profile and iron content make it highly susceptible to oxidation (Sabuncular et al. 2021). Extending shelf life by controlling these factors has become crucial for ensuring food safety and quality (Bilecen Şen and Kılıç 2021). Various preservation methods, applied alone or in combination, help maintain meat quality and safety (Mor‐Mur and Yuste 2010).

The use of edible films and coatings in food preservation has gained attention in recent years (Yong et al. 2021). These films and coatings, derived from natural materials, are biodegradable, nontoxic, and environmentally friendly (Chen et al. 2022; Singh et al. 2022). They can be enhanced with bioactive compounds, chemically modified biopolymers, cross‐linking agents, and plasticizers (Chen et al. 2022; Khaledian et al. 2024). Bioactive edible films and coatings can also integrate antioxidants, antimicrobials, colorants, flavorings, and nutraceuticals to improve functionality (Shahbazi and Shavisi 2018; Umaraw et al. 2020).

κ‐Carrageenan, an FDA‐approved food additive extracted from red algae, is widely used in the food industry for its stabilizing, thickening, gelling, emulsifying, and water‐retaining properties (Necas and Bartosikova 2013). Its reliability, sustainability, and accessibility make it a promising biopolymer for edible films and coatings (Udo et al. 2023).

Recent studies have shown that incorporating natural bioactive compounds into films and coatings enhances their antioxidant (Liu et al. 2015; Noor et al. 2018), antimicrobial (Maqsood et al. 2013; Panahi and Mohsenzadeh 2022), colorant (Wang et al. 2023), and nutritional properties (Yong et al. 2021), while preventing browning reactions (Santos et al. 2022) and improving protein cross‐linking (Benbettaieb et al. 2019).

Resveratrol (*trans*‐3,5,4ʹ‐trihydroxy‐stilbene), a polyphenol found in black grapes, peanuts, and blueberries, exhibits strong antioxidant properties and is effective in inhibiting lipid oxidation and extending food shelf life (Gülçin 2010; Salehi et al. 2018; Gündoğdu et al. 2021; Ansarian et al. 2022). It has been effectively incorporated into edible films and coatings for various meats (e.g., pork, fish) and processed meat products (e.g., meatballs, sausages) (Xiong et al. 2020; Bazargani‐Gilani and Pajohi‐Alamoti 2020; Hashemi et al. 2023).

Quercetin, a flavonol found in fruits and vegetables, offers antioxidant, anticancer, antiviral, antibacterial, and anti‐inflammatory benefits (Anand David et al. 2016; Batiha et al. 2020; Shabir et al. 2022). It enhances the properties of edible films, such as surface morphology, tensile strength, and opacity, making it suitable for active food packaging (Huang et al. 2020; Yadav et al. 2020; Roy and Rhim 2022; Shabir et al. 2022; Zeng et al. 2024).

Recent studies have demonstrated that bioactive edible films can significantly improve the quality and shelf life of goat meat (Smeti et al. 2021; Ghafari et al. 2024). Given the increasing demand for sustainable and natural preservatives in the food industry, this study aims to investigate the potential of resveratrol‐ or quercetin‐enriched κ‐carrageenan‐based edible films to improve the preservation and quality of goat meat burger patties. This research fills a significant gap in the literature by combining these bioactive compounds with κ‐carrageenan‐based films and evaluating their impact specifically on meat products. While previous studies have explored the effects of resveratrol or quercetin in various food applications, no study has yet examined their use in edible films for meat products, particularly goat meat. By assessing the antioxidant, antibacterial, and technological properties of these films, this research not only addresses the growing need for natural preservatives but also provides valuable insights into their practical application in enhancing the quality, safety, and sustainability of meat products. This research offers an important contribution by providing a sustainable and natural alternative to synthetic preservatives in the food industry, which is crucial for both improving food safety and meeting the growing consumer demand for clean‐label products.

## 2 Materials and Methods

### 2.1 Materials

Goat meat, 24 h postmortem, was purchased from a local meat company (Burdur, Türkiye). The goat meat was obtained from the hind legs of female goats with good conformation (slaughtered at approximately 1.5–2 years old and weighing 18–20 kg). Approximately 1–1.5 kg of meat was obtained from one hind leg, and a total of four legs were used.

All solvents used were of analytical and HPLC grade. The Folin–Ciocalteu reagent and sodium carbonate were purchased from Sigma–Aldrich. Methanol, ethanol, hydrochloric acid, and potassium chloride were supplied by Merck (Darmstadt, Germany). κ‐Carrageenan (CAS 9000‐07‐1), glycerol (CAS 56‐81‐5), resveratrol (CAS 501‐36‐0), and quercetin (CAS 117‐39‐5) were purchased from Sigma–Aldrich, USA. All culture media were obtained from Merck, Germany.

### 2.2 Preparation of κ‐Carrageenan‐Based Edible Films With Bioactive Compounds

**FIGURE 1:**
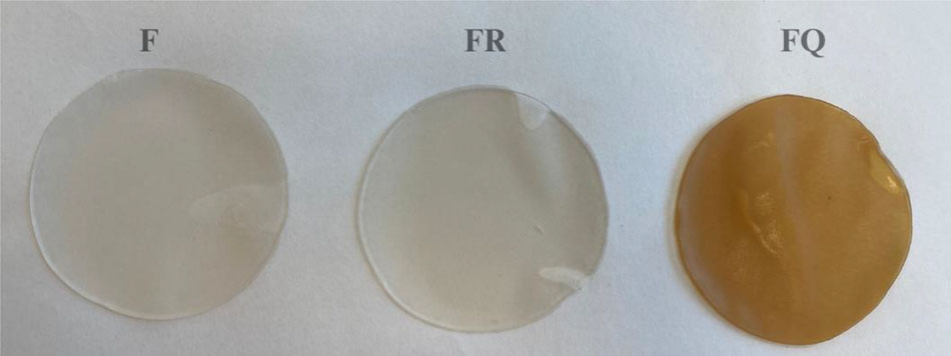
Appearance of the κ‐carrageenan‐based edible films (F: κ‐carrageenan‐based edible film without bioactive compounds; FR: κ‐carrageenan‐based edible film containing resveratrol; FQ: κ‐carrageenan‐based edible film containing quercetin).

The κ‐carrageenan‐based edible film solution was prepared with modifications to the method proposed by Velásquez et al. (2022), in two independent batches. κ‐Carrageenan (1% w/v) was dissolved in distilled water, followed by the incorporation of 1.5% (v/v) glycerol. The film solution was then exposed to heat at 90°C ± 2°C for 40 min while stirring consistently throughout the duration. After the heating process, 0.01% (w/v) sunflower oil and 0.2% (w/v) KCl were added to the solution. The sunflower oil was included to reduce the water vapor permeability of the films by introducing a hydrophobic component and to improve their flexibility, while KCl was used to enhance the mechanical strength by promoting intermolecular interactions. Subsequently, resveratrol or quercetin was added to the film solution based on the group contents. The control group (C) consisted of samples without any edible film application. The F group was coated with κ‐carrageenan‐based edible film without any bioactive compound. The FR group was treated with κ‐carrageenan‐based edible film containing 0.001% resveratrol, while the FQ group was coated with κ‐carrageenan‐based edible film containing 0.05% quercetin. The final concentrations (0.001% resveratrol and 0.05% quercetin) were determined not for direct equivalence but based on their individual efficacy as established in the literature and confirmed through preliminary tests, in order to ensure both functional performance and formulation stability (Souza et al. 2015; Silva‐Weiss et al. 2018; Ansarian et al. 2022; Abdalbeygi et al. 2022). Afterward, the solution was mixed. Briefly, 15 mL of each solution was transferred into plastic Petri dishes (100 × 15 mm), and the dishes were left to dry at 37°C for 24 h. Upon completion of the period, the films were removed from the Petri dishes and stored in a desiccator until the analyses were conducted (Figure 1). The thickness of the films was measured at five random points per film using a digital micrometer (Mitutoyo IP65, Mitutoyo Corp., Japan), and the average value was calculated. Since the variation among the measurements was minimal (±0.002 mm), the average value was reported as 0.1 mm for all treatments.

### 2.3 Analyses of Bioactive Compounds and κ‐Carrageenan‐Based Edible Films

#### 2.3.1 Total Phenolic Content (TPC) Analysis

The TPC analysis was performed with modifications to the method proposed by Singleton and Rossi (1965). From each sample, 40 µL was taken and 2.4 mL of distilled water was incorporated. The tubes were vortexed, and then 200 µL of Folin–Ciocalteu reagent and 600 µL of saturated sodium carbonate (Na_2_CO_3_) were added. The tubes were vortexed again, and the blend was stored in a dark environment at 20°C ± 2°C for 2 h. After, 760 µL of distilled water was transferred to the tubes, and the absorbance of the blue solution formed was quantified at a wavelength of 765 nm through a spectrophotometer (UV/VIS Spectrometer T80, PG instruments, UK). The TPC in the edible film solutions was determined as gallic acid equivalents (GAE) using a standard calibration curve.

#### 2.3.2 1,1‐Diphenyl‐2‐Picrylhydrazyl (DPPH) Radical Scavenging Activity

The DPPH radical scavenging activity (% inhibition) was evaluated with modifications to the method suggested by Dorman et al. (2003). From each sample, 50 µL was taken and 0.8 mL of methanol was incorporated. Then, 0.2 mL of a 1 mM DPPH radical solution prepared in methanol was incorporated to the tubes, and the tubes were vortexed. The blend was kept in the dark at 20°C ± 2°C for 30 min. Upon completion of the period, the absorbances were recorded at 515 nm with a spectrophotometer (UV/VIS Spectrometer T80, PG instruments, UK) against a control solution prepared with methanol and DPPH radical. The inhibition values (%) of the samples were determined with the formula below:

$$\mathrm{Inhibition}\left(\%\right)=\left(\left(\mathrm{Ab}s_{\mathrm{DPPH}}-\mathrm{Ab}s_{\mathrm{sample}}\right)/\mathrm{Ab}s_{\mathrm{DPPH}}\right)\times 100,$$

where Abs_DPPH_ is the absorbance of the DPPH solution and Abs_sample_ is the absorbance of the sample.

#### 2.3.3 Ferric Reducing Antioxidant Power (FRAP) Analysis

The FRAP content was determined with modifications to the method proposed by Benzie and Strain (1996). The FRAP reagent was made by mixing 300 mM/L acetate buffer, 10 mM/L TPTZ (2,4,6‐tri(2‐pyridyl)‐s‐triazine), and 20 mM/L ferric chloride hexahydrate (FeCl_3_·6H_2_O) in a 10:1:1 ratio. From each sample, 200 µL was taken and 3.8 mL of FRAP reagent was incorporated. The blend was left to stand in the dark at 20°C ± 2°C for 4 min and then read against a blank prepared with distilled water at a wavelength of 593 nm using a spectrophotometer (UV/VIS Spectrometer T80, PG instruments, UK). A standard calibration curve was generated using iron (II) sulfate heptahydrate (FeSO_4_·7H_2_O) at different concentrations (100–2000 µmol/L), and the FRAP content in the compounds and film solutions was calculated in µmol Fe^+2^/L.

#### 2.3.4 Water Solubility Analysis

The water solubility analysis is described as the percentage of dry matter of films that were immersed in water for 24 h. Samples were dried at 105°C for 24 h and then weighed to record their initial weight (*S*_0_). Then, 30 mL of distilled water was incorporated to the weighed samples, which were covered with parafilm and left at 20°C ± 2°C for 24 h. The insoluble portion was filtered, dried at the same temperature and time, and weighed (*S*_1_). The water solubility of the samples was computed using the formula below (Nair et al. 2011):

$$\mathrm{Solubility}\left(\%\right)=\left(\left(S_{0}-S_{1}\right)/S_{0}\right)\times 100,$$

where *S*_0_ is the initial weight of the films and *S*_1_ is the final weight of the films after drying.

#### 2.3.5 Color and Opacity Analysis

Konica Minolta color measurement device (Konica‐Minolta CR400, Osaka, Japan) was utilized to measure the lightness (CIE L^*^), redness (CIE a^*^), and yellowness (CIE b^*^) color values. The device, equipped using a D65 illuminant, an 8.0 mm aperture size, and a 10° standard observer angle, was calibrated against its own standard. Measurements were then taken from three different points on the film surface. For opacity measurement, the films were cut into rectangular shapes (1 × 4 cm) to fit the dimensions of the spectrophotometer cuvette and placed into the cuvettes. Absorbance values were read at a wavelength of 600 nm. The opacity values were calculated using the formula below (Friesen et al. 2015):

$$\mathrm{Opacity}=\mathrm{Ab}s_{600}/x,$$

where *x* is the thickness (mm) and Abs_600_ is the absorbance value at a wavelength of 600 nm.

#### 2.3.6 Determination of Antimicrobial Activity

The antimicrobial activities against *Listeria monocytogenes* (ATCC 19115), *Escherichia coli* (ATCC 25922), *Staphylococcus aureus* (ATCC 25923), and *Salmonella* Typhimurium (ATCC 14028) were assessed through the disk diffusion method (Ramos et al. 2012). These strains were obtained from the stock culture collection of the Food Technology Laboratory at Burdur Mehmet Akif Ersoy University (Burdur, Türkiye). Colonies from the stock culture were inoculated into Tryptic Soy Broth (CASO) medium. Following incubation at 37°C for 24 h, the intensity of the developed cultures was adjusted to 10^5^ CFU/mL using a McFarland device (Biosan DEN‐1 Densitometer, Riga, Latvia). Then, 0.1 mL of each culture was plated onto sterile Mueller–Hinton agar plates using a sterile swab. The prepared films were cut into 9‐mm‐diameter disks using a sterile punch and placed onto the agar plates with the surface inoculation. The Petri dishes were kept at 37°C for 24 h, after which the diameters (mm) of the clear zones around the film disks were recorded using a digital caliper (Vizbrite; accurate to 0.01 mm). Two replicate plates were used for each group, and the experiment was repeated twice.

#### 2.3.7 Scanning Electron Microscopy (SEM) Analysis

The surface images were acquired using a scanning electron microscope (FEI Quanta 250 FEG, Oregon, USA) at the Süleyman Demirel University YETEM‐Innovative Technologies Application and Research Center under low vacuum (10 kV). SEM images were taken at magnifications of 500× and 1000× to determine the microstructures of the samples.

### 2.4 Preparation of Goat Meat Burger Patties

The production of burger patties from goat meat was carried out in two independent batches. The amounts of water (10%) and salt (1%) were calculated based on the meat weight. The minced meat mixture was manually kneaded for approximately 20 min and portioned into equal amounts by chance (each patty dough was approximately 100 g). The patty dough was then shaped into round patties with a 10‐cm diameter and 1 cm thickness using a metal mold (16 patties for each replicate). Following shaping, the patties were randomly and equally assigned to each experimental group (C, F, FR, FQ). Cooking was carried out using an electric oven (MF 44; Arçelik, Istanbul, Türkiye), and to ensure even cooking of each patty, a digital oven thermometer (DT1004A; Cheerman Electronics Co., Ltd., Hong Kong, China) was used to cook the patties until the internal temperature reached 75°C. A total of four patties per group were analyzed across the storage period (one per time point).

### 2.5 Application of κ‐Carrageenan‐Based Edible Films on Goat Meat Burger Patties

The edible films were placed on the top and bottom surfaces of the burger patties, ensuring the whole surface of the patty was covered with the film. The patties were then stored in sterile low‐density polyethylene (LDPE) bags at 4°C for 14 days. The moisture, ash, fat, and protein content of the burger patties were evaluated on the production day (Day 0) and at the end of the storage period (Day 14). One patty (approximately 70 g after cooking) was used per group on both storage days in each of the two independent batches for all chemical composition analyses. Additionally, during the storage period, measurements were taken at specific intervals (Days 0, 1, 7, and 14) for pH, water activity, CIE L^*^, a^*^, and b^*^ color values thiobarbituric acid reactive substances (TBARS) analysis and counts of total mesophilic aerobic bacteria (TMAB), total coliform group bacteria, and mold–yeast.

### 2.6 Chemical Composition Analyses

Moisture content was determined using the oven‐drying method described in the Association of Official Agricultural Chemists (AOAC) Official Method 950.46 (AOAC International 1995). Ash content was measured according to the ashing procedure outlined in AOAC Official Method 923.03 (AOAC International 1995). Total lipid content was extracted using the method of Folch et al. (1957), in accordance with AOAC Official Method 983.23 (AOAC International 2006). Total nitrogen–protein content was determined using a Dumatherm device (Gerhardt GmbH & Co. KG, Königswinter, Germany) according to AOAC Official Method 968.06 (AOAC International 1990), and the percentage of protein was calculated by multiplying the nitrogen content by a factor of 6.25.

### 2.7 pH, Water Activity, and the Total Color Differences

During the storage period, the pH values were measured by bringing the samples to 20°C ± 2°C and inserting the pH meter (Testo 205, Lenzkirch, Germany) probe. Between each measurement, the electrode was cleaned with distilled water and the device was calibrated with pH 4.0–7.0 buffer solutions before analysis (Cemeroğlu 2010). Additionally, the pH value of the untreated raw goat meat was also determined prior to the experiment. The water activity (*a*_w_) values were measured using an *a*_w_ meter (Testo 645, Testo SE and Co. KGaA, Lenzkirch, Germany). The samples were reduced in size with a knife and placed into the sample chamber of the measurement block. The *a*_w_ values were determined at 20°C ± 2°C using equilibrium relative humidity. Color measurements were performed on the freshly cut inner surface of the samples in duplicates, with four surface readings taken from each sample using a color measurement device (Konica‐Minolta CR400, Osaka, Japan). The total color difference (ΔE^*^) values were determined based on the CIE L^*^, a^*^, and b^*^ color parameters, with the color values recorded immediately after removal from refrigerated storage to reflect their appearance prior to blooming. The instrument was adjusted to measure color using the L^*^a^*^b^*^ system with D65 illuminant, a 10° observer angle, and an 8.0 mm aperture size. Calibration of the colorimeter was performed using the white calibration plate (Bilecen Şen and Kılıç 2021).

### 2.8 TBARS Analysis

TBARS analysis was conducted in duplicates, following the method proposed by Kilic and Richards (2003). One gram of meat sample was taken, and 6 mL of trichloroacetic acid (TCA) solution containing EDTA and propyl gallate was added. The samples were homogenized using a homogenizer (Daihan Scientific Co. Ltd., WiseTis HG‐15D, Gang‐Won‐Do, South Korea) for 15 s and then filtered through filter paper (Whatman No. 1). From the obtained filtrate, 1 mL was taken, and 1 mL of thiobarbituric acid (TBA) was added and vortexed. Following 40 min of heating at 100°C, the mixture was cooled. Subsequent to cooling, the absorbance values of the samples were read using a spectrophotometer (UV/VIS Spectrometer T80, PG instruments, UK) against a control sample containing only 1 mL of TCA solution and 1 mL of TBA solution at a wavelength of 532 nm. The obtained absorbance values were multiplied by the coefficient derived from a prepared standard curve to determine the TBARS values of the samples in µmol/kg.

### 2.9 Microbiological Analyses

Ten grams of the sample was weighed sterilely, and 90 mL of sterile 0.85% sodium chloride solution (NaCl) was incorporated. The samples were then homogenized, and appropriate dilutions were prepared. From the prepared dilutions, 0.1 mL was transferred onto Plate Count Agar (PCA) for TMAB, Eosin Methylene Blue (EMB) for total coliform group bacteria, and Potato Dextrose Agar (PDA) for mold–yeast count using the spread plate method, and Petri dishes were transferred to the incubator at 30°C for 48 h, 37°C for 48 h, and 25°C for 5 days, respectively. After incubation, colonies between 15 and 300 were counted. Colony‐forming units (CFU) per gram were used to express the count results.

### 2.10 Statistical Analysis

The experiments applied to both the edible films and the goat meat burger patties were performed with two independent batches. Each batch included three different edible film treatments (F, FR, and FQ) and four different burger patty treatments (C, F, FR, and FQ). The chemical composition analyses of the patties were described using a General Linear Model (GLM), with storage days (0 and 14) and treatments (C, F, FR, and FQ) as fixed effects, and batch (two independent batches) as a random effect. pH, water activity, and TBARS data were analyzed in the same manner. The only change in the fixed effect for these analyses was the increase in storage days to include 0, 1, 7, and 14. Additionally, an interaction term for storage day × treatment was included in the model to analyze the interaction between these factors, and a two‐way analysis of variance (ANOVA) was conducted. Prior to the analysis, normality of residuals and homogeneity of variances were assessed using the Shapiro–Wilk and Levene's tests, respectively. The GLM was selected to accommodate both fixed and random effects, as well as potential interactions between fixed factors. Means and standard errors were derived from the models. Tukey's test for multiple comparisons of means was applied, and the critical value for a statistically significant effect was set at *p* < 0.05. Furthermore, the individual effects of each factor (storage days or treatment) were evaluated for statistical significance at *p* < 0.05 using one‐way ANOVA and Tukey's test. In addition, the TPC, antioxidant activity, water solubility, color, and opacity of the edible films were analyzed using one‐way ANOVA, with treatments (F, FR, and FQ) as fixed effects and batch (two independent batches) as a random effect. Comparison of means was performed using Tukey's test, with significance determined at the 0.05 level. All statistical models were constructed in Minitab 13 Statistical Software (Minitab Inc., State College, PA, USA).

## 3 Results and Discussion

### 3.1 TPC and Antioxidant Activity

The TPC (mg GAE/g), DPPH radical scavenging activity (% inhibition), and FRAP (µmol Fe^+2^/L) results for the bioactive compounds and edible film solutions are presented in Table 1. The highest TPC was observed in the film solution without bioactive compounds (F), while the lowest TPC was found in the resveratrol‐containing film (FR) (*p* < 0.05). This outcome may be related to the known limitations of the Folin–Ciocalteu assay, which is not entirely specific to phenolic compounds and can react with other reducing substances such as polysaccharides, sugars, and proteins (Prior et al. 2005). κ‐Carrageenan itself has antioxidant properties (Yuan et al. 2006), which may have contributed to the unexpectedly high TPC in the control group. In contrast, the lower TPC values in the films containing bioactive compound may be a result of interactions between resveratrol or quercetin and the film matrix or the partial degradation of these compounds during film preparation, which may limit their detection through the assay.

**TABLE 1:** Total phenolic contents (TPC) (mg GAE/g), DPPH radical scavenging activity (% inhibition), and FRAP (µmol Fe^+2^/L) results of the bioactive compounds and edible film solutions.

	Treatment					
Parameter	R	Q	F	FR	FQ	Significance
TPC (mg GAE/g)	n.a.	n.a.	405.43 ± 0.48^a^	149.71 ± 0.24^c^	389.00 ± 0.42^b^	*
DPPH (% inhibition)	89.72 ± 0.05^b^	95.19 ± 0.01^a^	27.90 ± 0.01^c^	7.44 ± 0.00^d^	91.85 ± 0.06^ab^	*
FRAP (µmol Fe^+2^/L)	204.31 ± 0.57^c^	1757.77 ± 0.31^a^	148.92 ± 0.45^c^	171.23 ± 0.38^c^	545.08 ± 0.37^b^	*

*Note*: Means with different letters in a row differ statistically significantly at *p*‐value; *p < 0.05. The results are presented as mean ± standard error. n.a.—not analyzed; as pure resveratrol and quercetin were utilized, the TPC analysis was not performed on these compounds (R: resveratrol; Q: quercetin; F: κ‐carrageenan‐based edible film solution without bioactive compounds; FR: κ‐carrageenan‐based edible film solution containing resveratrol; FQ: κ‐carrageenan‐based edible film solution containing quercetin).

Quercetin‐containing film (FQ) showed higher TPC than resveratrol‐containing film (FR) (*p* < 0.05), supporting previous studies where quercetin maintained its antioxidant properties in various matrices. Quercetin's antioxidant activity is attributed to mechanisms such as free metal binding, free radical scavenging, and enzyme modulation (Yalçın et al. 2017). In contrast, resveratrol also exhibits antioxidant potential (Carrizzo et al. 2013; Gambini et al. 2015; Sarman and Gülle 2021), but its stability is more sensitive to processing conditions. Studies have shown that high temperatures can cause degradation of resveratrol, which may explain the lower TPC in the FR group (Meral 2016).

However, previous studies have shown that bioactive extracts can increase TPC in κ‐carrageenan films (Farhan and Hani 2020; Jancikova et al. 2020; Velásquez et al. 2022; Gumus et al. 2024). This contradiction highlights the complex behavior of phenolic compounds during film preparation, where factors such as temperature, drying duration, and solvent composition can affect stability (Meral 2016). The high processing temperature in this study likely contributed to the reduced TPC in both resveratrol and quercetin films, aligning with previous research that has noted a reduction in TPC with high heat treatment (Menzek 2023). In conclusion, the present study's findings on TPC, particularly the lower values observed with resveratrol, are consistent with previous research indicating that the stability of bioactive compounds is highly dependent on processing conditions. However, the unexpected high TPC in the control group and the differences between quercetin and resveratrol emphasize the need for further exploration of the interactions between bioactive compounds and the film matrix, as well as the impact of processing parameters on compound stability.

The inhibition value of quercetin (Q) was higher than that of resveratrol (R) (*p* < 0.05). Both resveratrol and quercetin exhibit strong antioxidant properties (Duenas et al. 2010; Sueishi et al. 2017), with quercetin showing superior activity in DPPH radical scavenging (Bardakçı Yılmaz and Boyacıoğlu 2020). The inhibition value of resveratrol‐containing film (FR) was significantly lower than that of free resveratrol (R) (*p* < 0.05), likely due to resveratrol's instability under high temperatures, with its activity decreasing by approximately 82%. This result aligns with previous studies indicating that resveratrol can degrade under environmental stressors such as high temperature and oxygen exposure (Fiod Riccio et al. 2020; Bancuta et al. 2018). Although some studies suggest that resveratrol enhances antioxidant activity in edible films (Ansarian et al. 2022; Hashemi et al. 2023), in this study, the film preparation process appeared to negatively affect resveratrol's stability. In contrast, no significant difference was found between the inhibition values of quercetin (Q) and the quercetin‐containing film (FQ) (*p* > 0.05), suggesting that quercetin remained stable and retained its antioxidant activity during film preparation.

The FRAP analysis revealed that quercetin (Q) exhibited the highest FRAP value (*p* < 0.05). Known for its potent antioxidant properties, quercetin is a major polyphenols found in plant‐based foods, with strong antioxidant effects demonstrated in several studies (Gordon and Penman 1998; Jullian et al. 2007; Lesjak et al. 2018; Xu et al. 2019). Duenas et al. (2010) found that quercetin outperforms catechin, epicatechin, their methylated derivatives, and tocopherol in antioxidant activity. In this study, quercetin showed the highest antioxidant capacity with a FRAP value of 1757.77 ± 0.31 (µmol Fe^+2^/L) (*p* < 0.05). No significant difference was observed between the FRAP values of resveratrol (R) and its film solution (FR) (*p* > 0.05). However, the FRAP value of the quercetin‐containing film (FQ) was significantly lower than that of free quercetin (Q) (*p* < 0.05). Similar result was reported by de Albuquerque et al. (2023), who found that free quercetin exhibited higher antioxidant activity than quercetin‐loaded lecithin liposomes.

### 3.2 Water Solubility, Color, and Opacity Analysis

Table 2 shows the water solubility, CIE L^*^, a^*^, and b^*^ color values, and opacity of the edible films. Water solubility plays a crucial role in determining the shelf life of packaged food (Kim et al. 2002). Polysaccharide‐based edible films are typically hydrophilic, leading to higher water solubility. In this study, no significant differences in water solubility were observed among the treatments (*p* > 0.05). The addition of resveratrol or quercetin did not significantly affect water solubility, indicating minor changes in the film's hydrophilic properties (Perez‐Gago et al. 2006).

**TABLE 2:** Water solubility, CIE L^*^, a^*^, and b^*^ color values, and opacity of the edible films.

		Treatment			
Parameter		F	FR	FQ	Significance
Water solubility		55.64 ± 1.06^a^	52.58 ± 0.94^a^	56.30 ± 1.28^a^	n.s.
Color values	L^*^	91.7 ± 0.26^a^	89.1 ± 0.34^b^	66.9 ± 0.70^b^	*
	a^*^	0.1 ± 0.03^b^	0.2 ± 0.04^b^	7.5 ± 0.34^a^	*
	b^*^	4.9 ± 0.18^c^	6.1 ± 0.13^b^	39.5 ± 0.11^a^	*
Opacity		4.55 ± 0.37^b^	2.33 ± 0.24^c^	9.21 ± 0.19^a^	*

*Note*: Means with different letters in a row differ statistically significantly at *p*‐value; *p < 0.05. The results are presented as mean ± standard error. n.s.—not significant (F: κ‐carrageenan‐based edible film without bioactive compounds; FR: κ‐carrageenan‐based edible film containing resveratrol; FK: κ‐carrageenan‐based edible film containing quercetin).

For color values, no significant difference was found between the L^*^ values of films containing resveratrol (FR) and quercetin (FQ) (*p* > 0.05). However, the film without bioactive compounds (F) showed the highest L^*^ value. The a^*^ and b^*^ values of the quercetin‐containing film (FQ) were significantly higher than those of the other films (F and FR) (*p* < 0.05), indicating that quercetin influenced the color more than resveratrol. The addition of resveratrol decreased the L^*^ value (*p* < 0.05), had no significant effect on the a^*^ value (*p* > 0.05), and increased the b^*^ value (*p* < 0.05). Quercetin, in contrast, increased both a^*^ and b^*^ values (*p* < 0.05). These changes in color are attributed to quercetin's natural pigments and polyphenols (Giteru et al. 2015; Figure 1). Sutharsan et al. (2022) found similar results, with quercetin increasing b^*^ values in chitosan‐based films. Wu et al. (2023) reported that resveratrol added to starch–gelatin films enhanced both a^*^ and b^*^ values and reduced L^*^ values.

Regarding opacity, resveratrol decreased the opacity, while quercetin increased it (*p* < 0.05). The higher opacity values in the quercetin film are attributed to the color pigments and interactions between phenolic compounds, which reduce light transmission and increase scattering (Jakubowska et al. 2023).

### 3.3 Determination of Antimicrobial Activity

The κ‐carrageenan‐based edible films did not show any inhibition zones against *L. monocytogenes*, *E. coli*, *S. aureus*, or *S*. Typhimurium. Some studies suggest that Gram‐negative bacteria exhibit greater resistance to phenolic compounds compared to Gram‐positive bacteria. This resistance is due to the outer lipopolysaccharide membrane, which restricts the release of hydrophobic components. In contrasts, Gram‐positive bacteria, with their double‐layered phospholipid membranes, interact more readily with the hydrophobic components of phenolic compounds. This interaction increases ion permeability, leading to the leakage of essential cell components and disruption of bacterial enzyme systems, ultimately resulting in cell death (Sandri et al. 2007).

In this study, it is hypothesized that the antimicrobial activity of the bioactive compounds may be reduced due to their breakdown by hydrolytic and detoxifying enzymes in the periplasmic space of the bacteria. Previous studies have also indicated that antimicrobial activity can vary depending on factors such as the bacterial strain, the concentration of the bioactive compound, and its structure (Zamuz et al. 2021). For instance, Sanla‐Ead et al. (2012) found that cellulose‐based films containing cinnamaldehyde or eugenol did not produce inhibition zones against *S*. Enteritidis, *E. coli*, *E. coli* O157:H7, *S. aureus*, *L. monocytogenes*, *Aeromonas hydrophila*, *Bacillus cereus*, *Micrococcus luteus*, and *Pseudomonas aeruginosa*, likely due to the low solubility of the bioactive compounds and the absence of emulsifiers.

### 3.4 SEM Analysis

**FIGURE 2:**
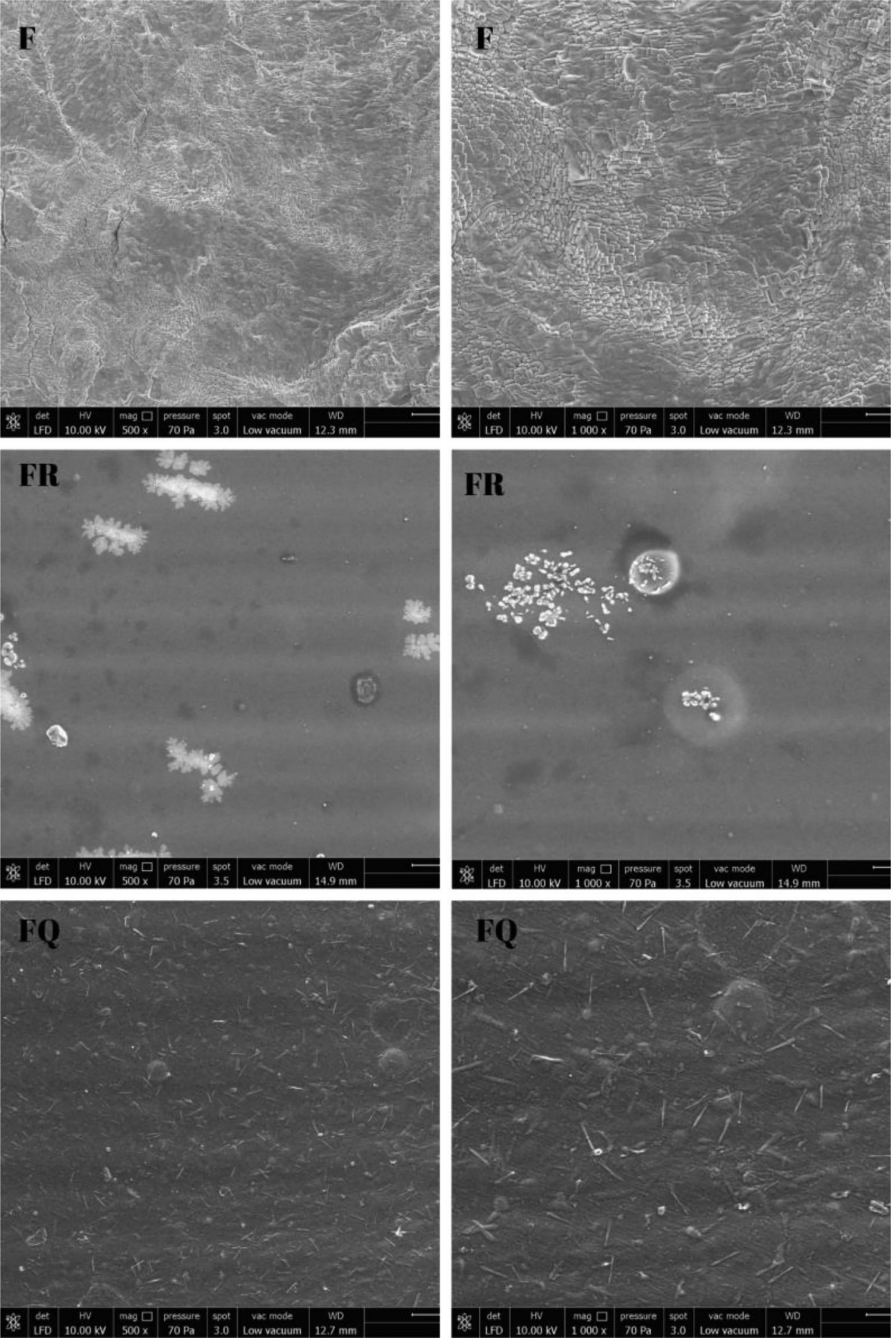
Surface SEM micrographs of the κ‐carrageenan‐based edible films (F: κ‐carrageenan‐based edible film without bioactive compounds; FR: κ‐carrageenan‐based edible film containing resveratrol; FQ: κ‐carrageenan‐based edible film containing quercetin; left column 500× magnification, right column 1000× magnification).

Surface images of κ‐carrageenan‐based edible films at 500× and 1000× magnifications are presented in Figure 2. SEM micrographs revealed that the incorporation of quercetin into the film solution (FQ) resulted in a relatively homogeneous distribution within the film matrix. In contrast, the distribution of resveratrol in the film (FR) appeared to be less uniform, likely due to its limited solubility in water and other polar solvents. As a more apolar compound, resveratrol exhibits lower solubility, which can hinder its even dispersion within hydrophilic film matrices. Additionally, external factors such as pH and temperature, along with its molecular structure, may further influence its distribution pattern.

Furthermore, the film without bioactive compounds (F) exhibited visible cracks and discontinuities on its surface, whereas the film containing resveratrol (FR) displayed a smoother and more continuous morphology. This finding is consistent with the observations of Farhan and Hani (2017), who reported rough and branched surface structures in semirefined κ‐carrageenan films, attributing this to strong intermolecular interactions within the κ‐carrageenan matrix.

The film containing quercetin (FQ) also exhibited fewer air bubbles and cracks compared to the film without bioactive compounds (F). The results align with the findings of Huang et al. (2020), who reported that the addition of quercetin to kefiran‐based films improved surface smoothness due to the formation of new bonds that stabilized the polymer network. Similarly, in the present study, both FR and FQ films showed more structurally integrated and homogeneous matrices compared to the F films, indicating improved film integrity.

The surface characteristics of edible films, including smoothness and homogeneity, are critical for their functionality in food coating and packaging. Bharti et al. (2021) also observed fine structural integrity in sweet potato starch and κ‐carrageenan‐based films. In contrast, Pastor et al. (2013) reported that resveratrol incorporation into chitosan films led to increased surface roughness, potentially due to the formation of complex intermolecular bonds caused by stilbene structures. These findings suggest that while both resveratrol and quercetin can enhance certain structural properties of edible films, their effects vary depending on their chemical nature and compatibility with the film matrix.

### 3.5 Chemical Composition

The protein content of the burger patties was not significantly affected by treatments, storage days, or their interaction (*p* > 0.05; Table 3). However, storage day alone had a statistically significant effect (*p* < 0.05) on the moisture, ash, and fat contents of the patties, whereas treatments and their interaction did not show any significant influence (*p* > 0.05). By the end of the 14‐day storage period, a significant increase was observed in both ash and moisture contents, while fat content decreased significantly (*p* < 0.05).

**TABLE 3:** Impact of treatment and storage day on the moisture, ash, fat, and protein contents (%) of goat meat burger patties.

		Treatment					Significance		
Chemical composition	Day	C	F	FR	FQ	Overall means (day)	Treatment	Day	Interaction
Moisture (%)	0	59.38	60.48	59.92	59.76	59.88 ± 0.62^y^	n.s.	*	n.s.
	14	61.03	63.93	63.66	64.34	63.24 ± 0.53^x^			
	Overall means (treatment)	60.21 ± 0.73	62.20 ± 1.09	61.79 ± 0.93	62.05 ± 1.25				
Ash (%)	0	5.16	4.89	5.34	5.58	5.24 ± 0.21^y^	n.s.	*	n.s.
	14	5.49	6.63	7.50	7.42	6.76 ± 0.36^x^			
	Overall means (treatment)	5.33 ± 0.21	5.76 ± 0.72	6.42 ± 0.64	6.50 ± 0.54				
Fat (%)	0	28.62	28.55	28.04	28.96	28.54 ± 0.23^x^	n.s.	*	n.s.
	14	24.95	25.04	24.61	25.59	25.05 ± 0.37^y^			
	Overall means (treatment)	26.78 ± 0.93	26.79 ± 0.72	26.32 ± 0.78	27.28 ± 0.71				
Protein (%)	0	35.95	32.97	33.27	36.09	34.57 ± 0.69	n.s.	n.s.	n.s.
	14	33.6	33.71	35.00	33.67	34.00 ± 0.95			
	Overall means (treatment)	34.78 ± 1.80	33.34 ± 1.19	34.14 ± 0.66	34.88 ± 0.98				

*Note*: Means with different letters in a column differ statistically significantly at *p*‐value (significance of main factors—storage day); *p < 0.05. The results are presented as mean ± standard error. n.s.—not significant (C: control group without edible film application; F: group coated with κ‐carrageenan‐based edible film without bioactive compounds; FR: group coated with κ‐carrageenan‐based edible film containing resveratrol; FQ: group coated with κ‐carrageenan‐based edible film containing quercetin).

The chemical composition of goat meat is known to be affected by various factors, including breed (Ivanović et al. 2014), age (Wattanachant et al. 2008), gender, productivity, stress, environmental conditions (Sen et al. 2004), management practices, nutrition, slaughter weight, health status, and the pre‐ and post‐slaughter procedures applied to the carcasses (Paleari et al. 2008). Due to the wide range of influencing variables, comparing the chemical composition of goat meat across different studies is inherently challenging, as each study is conducted under distinct conditions that may yield varying results.

Furthermore, several studies have reported that the application of edible films and coatings does not significantly affect the chemical composition of meat and meat products. For example, the moisture content of chicken nuggets and turkey meat was not significantly altered by maltodextrin‐ and alginate‐based edible films containing Indian ginseng (Sharma et al. 2021) or gelatin‐chitosan based films containing essential oil from *Ferulago angulata* (Naseri et al. 2020), respectively. Similarly, Dehghan Tanha et al. (2021) found that the ash content of fish sausage samples coated with a gelatin‐based solution containing purslane remained stable throughout storage. Additionally, Bilecen Şen and Kılıç (2021) reported that the fat content of meatballs was not influenced by the application of edible coatings.

### 3.6 pH, Water Activity, and Total Color Differences

The impact of treatment and storage day on the pH and *a*_w_ values of goat meat burger patties is shown in Table 4. The pH values of the patties were significantly influenced (*p* < 0.05) by the treatments and the interaction between treatment and storage days, while storage days alone had no significant effect (*p* > 0.05). At Day 0, the pH values ranged from 6.21 to 6.23 for all treatments. On Day 1, no significant differences in pH values were observed among the groups (*p* > 0.05). However, by Day 7, patties coated with resveratrol and quercetin films (FR and FQ) exhibited significantly lower pH values than the control group (C) (*p* < 0.05). This decrease may be attributed to the bioactive compounds in the films, which may help reduce surface microbial contamination, similar to findings by Bojorges et al. (2020) and Hamann et al. (2022). By Day 14, the pH value of the control group (C) increased, surpassing that of the quercetin‐coated patties (FQ) (*p* < 0.05). This rise in pH is likely due to the production of ammonia and amines from microbial activity in the meat, as suggested by Cortez‐Vega et al. (2012). In line with this, the control group had a higher microbial count (TMAB: 4.18 log_10_ CFU/g), while no microbial presence was observed in the quercetin‐coated group (<10 CFU/g) (as detailed in Section 3.8). This suggests that the microbial activity in the control group likely contributed to the observed pH increase. Moreover, no significant differences were observed in pH values between the bioactive component‐free film (F) and resveratrol film (FR) (*p* > 0.05). These findings suggest that bioactive films, particularly those containing quercetin, may help maintain lower pH values in patties, potentially extending shelf life and preserving food quality. This is consistent with previous studies, such as those by Bojorges et al. (2020), Khan et al. (2020), and Hamann et al. (2022), which noted similar pH trends in meats treated with antimicrobial edible films. In contrast, Yahaya et al. (2019) observed no significant changes in pH values of burger patties over a 14‐day storage period, which could be attributed to different experimental conditions or types of edible films used in their study. Nevertheless, the application of bioactive films may still provide a promising method for controlling pH levels and microbial growth, which could contribute to extending the shelf life and improving the quality of meat products.

**TABLE 4:** Impact of treatment and storage day on the pH, water activity (*a*_w_), and TBARS (µmol MDA/kg) values of goat meat burger patties.

		Treatment					Significance		
Parameter	Day	C	F	FR	FQ	Overall means (day)	Treatment	Day	Interaction
pH	0	6.21^cd^	6.22^bcd^	6.23^a–d^	6.23^a–d^	6.22 ± 0.02	*	n.s.	*
	1	6.21^cd^	6.23^a–d^	6.21^cd^	6.22^bcd^	6.21 ± 0.01			
	7	6.34^ab^	6.25^abc^	6.21^cd^	6.16^cd^	6.24 ± 0.02			
	14	6.35^a^	6.27^abc^	6.25^a–d^	6.12^d^	6.24 ± 0.02			
	Overall means (treatment)	6.27 ± 0.03^x^	6.24 ± 0.01^xy^	6.22 ± 0.01^yz^	6.18 ± 0.01^z^				
*a*_w_	0	0.846	0.886	0.911	0.911	0.888 ± 0.013	n.s.	n.s.	n.s.
	1	0.874	0.900	0.894	0.900	0.892 ± 0.006			
	7	0.877	0.880	0.899	0.905	0.890 ± 0.016			
	14	0.941	0.914	0.901	0.899	0.914 ± 0.008			
	Overall means (treatment)	0.884 ± 0.015	0.895 ± 0.013	0.901 ± 0.010	0.904 ± 0.009				
TBARS (µmol MDA/kg)	0	12.68^f^	12.19^f^	11.41^f^	11.40^f^	11.92 ± 0.58^z^	*	*	*
	1	20.17^g^	20.68^g^	21.04^g^	18.69^g^	20.14 ± 0.31^y^			
	7	33.99^bc^	32.18^cd^	28.61^de^	30.39^cde^	31.29 ± 0.56^x^			
	14	41.47^a^	33.46^bc^	36.44^b^	27.44^e^	34.70 ± 1.33^w^			
	Overall means (treatment)	27.08 ± 1.93^x^	24.62 ± 1.27^y^	24.38 ± 1.46^y^	21.98 ± 1.94^z^				

*Note*: Means with different letters (a–g) differ statistically significantly at *p*‐value (significance of treatment × storage day interaction). Means with different letters (w–z) in a row or column differ statistically significantly at *p*‐value (significance of main factors—treatment or storage day); *p < 0.05. The results are presented as mean ± standard error. n.s.—not significant (C: control group without edible film application; F: group coated with κ‐carrageenan‐based edible film without bioactive compounds; FR: group coated with κ‐carrageenan‐based edible film containing resveratrol; FQ: group coated with κ‐carrageenan‐based edible film containing quercetin).

The treatments, storage days, and their interaction did not result in any statistically significant differences in the water activity (*a*_w_) values of the patties (*p* > 0.05). On Day 0, the *a*_w_ values ranged from 0.846 to 0.911, increasing to a range of 0.899–0.941 by Day 14. These findings indicate that the application of edible films had no significant effect on the water activity of goat meat burger patties throughout the storage period.

**FIGURE 3:**
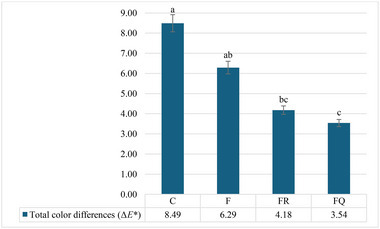
Total color differences (ΔE^*^) of goat meat hamburger patties during storage period (C: control group without edible film application; F: group coated with κ‐carrageenan‐based edible film without bioactive compounds; FR: group coated with κ‐carrageenan‐based edible film containing resveratrol; FQ: group coated with κ‐carrageenan‐based edible film containing quercetin).

The total color differences (ΔE^*^) of goat meat burger patties during the storage period are presented in Figure 3. The ΔE^*^ value of the patties coated with the resveratrol or quercetin films (FR or FQ) were significantly lower compared to the control group (C) (*p* < 0.05). However, no significant changes were detected between the ΔE^*^ values of the control group (C) and the patties coated with the bioactive component‐free film (F) (*p* > 0.05). These results suggest that the incorporation of resveratrol or quercetin into the film solution, rather than the application of the edible film alone, helped to maintain the initial color values of the burger patties for a longer period. It is likely that the polyphenolic compounds in the film solutions contributed to preserving the color of the patties due to their antioxidant and antimicrobial properties (Bilecen Şen and Kılıç 2021).

### 3.7 TBARS

The treatments, storage days, and their interaction significantly affected the TBARS values of burger patties (*p* < 0.05), as shown in Table 4. The TBARS values of the patties generally increased over the 14‐day storage period at 4°C (*p* < 0.05). No significant differences were observed in the TBARS values among the treatments on Days 0 and 1 (*p* > 0.05). However, on Day 7, the TBARS value of the patty coated with resveratrol film (FR) was significantly lower compared to the control group (C) (*p* < 0.05). On Day 14, the control group (C) exhibited the highest TBARS value, while the lowest TBARS value was observed in the patty coated with quercetin film (FQ) (*p* < 0.05).

The observed decrease in TBARS values in patties coated with quercetin‐enriched films compared to the control group suggests that the high antioxidant capacity of quercetin (91.85 ± 0.06% DPPH inhibition and 545.08 ± 0.37 µmol Fe⁺^2^/L FRAP) effectively delayed lipid oxidation. This inverse relationship between antioxidant capacity and lipid oxidation indicates a strong correlation between the film's phenolic content and its protective effect against oxidative spoilage. Furthermore, the TBARS values of patties coated with the bioactive component‐free film (F) and the resveratrol film (FR) showed no significant differences (*p* > 0.05).

The addition of bioactive compounds, especially quercetin, to the film solutions was found to delay lipid oxidation in the burger patties, and even the edible film alone helped prevent lipid oxidation. This antioxidant effect is attributed to the film creating a barrier between oxygen and the meat, thus delaying the myoglobin oxidation process (He and Wang 2022). Similar studies have also reported that the addition of bioactive compounds to edible films or coatings delayed lipid oxidation in pork patties (Qin et al. 2013), beef patties (Bilecen Şen and Kılıç 2021), camel meat (Ansarian et al. 2022), fresh sausages (Hamann et al. 2022), and cooked meat product (Bilecen Şen and Güleç 2024). He and Wang (2022) noted that the κ‐carrageenan coatings embedded with cinnamon essential oil reduced lipid oxidation and had the potential to prolong the shelf life of pork meat. Additionally, Wang et al. (2023) found that films made from arrowhead starch, κ‐carrageenan, and black chokeberry extract applied to chicken wings decreased lipid oxidation, suggesting that such films could be used in active and intelligent packaging in the food industry. Goudarzi et al. (2023) observed that k‐carrageenan–PVA electrospun fiber films incorporating *Prunus domestica* anthocyanins and epigallocatechin gallate improved the antioxidant properties of minced beef, supporting our findings.

The current results showed that the TBARS value of the patty coated with the quercetin film (FQ) was significantly lower than that of the patty coated with the resveratrol film (FR) (*p* < 0.05). This finding suggests that the antioxidant capacity of resveratrol may have been diminished by exposure to high temperatures, thereby reducing its effectiveness. Consequently, lipid oxidation occurred more rapidly in burger patties coated with resveratrol film compared to those coated with quercetin film. Quercetin's antioxidant activity has been shown to slow down the lipid oxidation process by eliminating free radicals in the aqueous phase (Souza et al. 2015). Furthermore, incorporating quercetin into biopolymers such as zein (Arcan and Yemenicioğlu 2011), chitosan (Jakubowska et al. 2023), chitosan–gelatin (Roy and Rhim 2022), kafirin (Huang et al. 2020), and carboxymethyl cellulose (Silva‐Weiss et al. 2018) has significantly enhanced the antioxidant properties of the resulting films.

Resveratrol is known to protect low‐density lipoproteins from peroxide degradation through both free radical scavenging and chelation mechanisms (Marinova et al. 2002). Due to its low resistance to light, resveratrol oxidizes quickly, which may explain why it becomes more potent when integrated into edible films or coatings (Busolo and Lagaron 2015; Martinez et al. 2018). Ansarian et al. (2022) found that nanoemulsion‐based basil seed gum edible films with clove and resveratrol essential oil reduced TBARS values in meats and delayed lipid oxidation. Similarly, Hashemi et al. (2023) observed that sodium alginate‐based films with thymol and resveratrol, particularly at the highest resveratrol concentration, resulted in the lowest TBARS values in cooked sausages.

### 3.8 Microbiological Analyses

In raw goat meat, the TMAB, total coliform bacteria, and mold–yeast counts were determined as 4.95, 3.78, and 3.60 log_10_ CFU/g, respectively. After the cooking process, microbial counts in both the patties and the edible film solution dropped below detectable limits (<10 CFU/g). Throughout the 14‐day storage period at 4°C, only the patties coated with the quercetin film (FQ) maintained TMAB levels below 10 CFU/g (*p* > 0.05). On Day 14, TMAB counts were 4.18, 3.55, and 3.29 log_10_ CFU/g for the control (C), bioactive‐free film (F), and resveratrol film (FR) groups, respectively (data not shown). These findings indicate that incorporating quercetin into edible film solution was more effective than resveratrol in inhibiting microbial growth during storage.

The superior antimicrobial activity of quercetin, coupled with its thermal stability, likely contributed to this result. The antimicrobial effect observed in the TMAB counts highlights the films’ broader potential in controlling microbial growth during storage, even though they did not directly inhibit specific pathogens in vitro. This suggests that the films, particularly the quercetin‐coated one, were effective in limiting overall microbial growth over time, despite differing from pathogen‐specific inhibition. Furthermore, the inhibition of TMAB growth in the quercetin‐coated group, along with the pH reduction (pH 6.18 ± 0.01 in the quercetin group compared to pH 6.27 ± 0.03 in the control group), suggests that quercetin's antimicrobial effect, combined with its ability to lower pH, helped prevent microbial growth and extend shelf life.

The highest TMAB count was found in the control group (C) (*p* < 0.05). TMAB are known contributors to spoilage in meat products, negatively impacting sensory quality (Siripatrawan and Noipha 2012). The application of edible films significantly inhibited TMAB growth in burger patties. In support of this, Xiong et al. (2020) demonstrated that pectin‐based edible coatings containing thyme essential oil and resveratrol nanoemulsion reduced microbial growth in pork fillets. The enhanced antimicrobial efficacy observed in this study is largely attributed to quercetin, which has been shown to inhibit Gram‐positive and Gram‐negative bacteria, fungi, and viruses via multiple mechanisms. These include the disruption of the cell wall and membrane, inhibition of protein and nucleic acid synthesis, suppression of virulence factor expression and enzyme activity, and prevention of biofilm formation (Veiko et al. 2023).

Several studies have confirmed the antimicrobial and antifungal properties of quercetin (Torres et al. 2012; Yadav et al. 2020). Bali et al. (2018) reported that among various phytochemicals tested, quercetin exhibited the strongest antimicrobial activity against foodborne pathogens. Furthermore, its incorporation into edible film and coating matrices has been widely employed to enhance antimicrobial efficacy (Olewnik‐Kruszkowska et al. 2021; Du et al. 2022; Zeng et al. 2024).

During the 14‐day storage at 4°C, both the total coliform and mold–yeast counts remained below 10 CFU/g (data not shown). This result is attributed to the internal temperature of patties reaching 75°C during cooking, exceeding the thermal death points of these microorganisms. Cooking is well‐documented as an effective microbial control strategy in meat products, ensuring microbiological safety (Jamwal et al. 2015; Noor et al. 2018).

## 4 Conclusions

Both quercetin and resveratrol demonstrated notable antioxidant capacities; however, the effectiveness of resveratrol appeared to be diminished under high‐temperature conditions due to its thermal sensitivity. Incorporating these compounds into κ‐carrageenan‐based edible films influenced the film's optical properties by reducing L^*^ values and increasing b^*^ values. While resveratrol led to a decrease in opacity, quercetin enhanced film opacity. Neither compound exhibited direct antimicrobial activity against *L. monocytogenes*, *E. coli*, *S. aureus*, and *S. Typhimurium* in vitro. However, SEM micrographs revealed that films containing resveratrol or quercetin formed more homogeneous and structurally intact matrices compared to the bioactive component‐free film.

The chemical compositions and water activity of the goat meat burger patties remained unaffected by the treatment, storage day, or their interaction. At the end of 14 days of refrigerated storage, patties coated with quercetin films showed significantly lower pH values than the control, alongside improved color retention. Furthermore, both bioactive films, particularly those containing quercetin, were effective in delaying lipid oxidation. Notably, the quercetin‐coated group maintained a TMAB count of <10 CFU/g, while the control exhibited the highest microbial load. Overall, the key finding of this study is that κ‐carrageenan‐based edible films enriched with quercetin can significantly extend the shelf life of heat‐treated ready‐to‐eat (RTE) meat products by delaying oxidative spoilage and suppressing microbial growth. These results support the potential of quercetin‐containing films as a sustainable, natural alternative to synthetic preservatives. Future research should explore the optimization of bioactive compound combinations and the development of advanced active packaging technologies, such as encapsulation and controlled‐release systems, to further enhance the preservation and sustainability of meat and meat products.

## 5 Limitations

This study presents several limitations that should be considered when interpreting the findings. First, the feasibility of scaling up the use of edible films in large‐scale meat production remains uncertain, as the current study was conducted at a laboratory scale. Additionally, the variability in meat sources, such as different animal breeds or production conditions, may affect the generalizability of the results. Furthermore, real‐world packaging conditions, such as storage time, temperature, and humidity, were not addressed in this study, and their impact on film performance remains unclear. Future research should aim to investigate these factors to evaluate the practical applicability of these edible films in commercial settings.

## Author Contributions

**Ezgi Şengül**: conceptualization, formal analysis, investigation, methodology, writing–original draft. **Damla Bilecen Şen**: conceptualization, formal analysis, investigation, methodology, supervision, writing–original draft, writing–review and editing, project administration.

## Conflicts of Interest

The authors declare no conflicts of interest.
